# Differences between echocardiography and cardiac nuclear magnetic resonance parameters in children with bicuspid aortic valve-related aortopathy

**DOI:** 10.3389/fcvm.2024.1384707

**Published:** 2024-11-26

**Authors:** Stasa Krasic, Boris Zec, Vesna Topic, Sasa Popovic, Dejan Nesic, Marija Zdravkovic, Vladislav Vukomanovic

**Affiliations:** ^1^ Cardiology Department, Mother and Child Health Institute of Serbia, Belgrade, Serbia; ^2^ School of Medicine, University of Belgrade, Belgrade, Serbia; ^3^ Pediatric Clinic, University Clinical Centre of the Republic of Srpska, Banja Luka, Republic of Srpska; ^4^ Radiology Department, Mother and Child Health Institute of Serbia, Belgrade, Serbia; ^5^ Faculty of Medicine, Institute of Medical Physiology, University of Belgrade, Belgrade, Serbia; ^6^ Cardiology Department, Clinical Hospital Center Bezanijska Kosa, Belgrade, Serbia

**Keywords:** bicuspid aortic valve, aortopathy, children, echocardiography, cardiac magnetic resonance

## Abstract

**Objectives:**

The bicuspid aortic valve (BAV) is the most common congenital heart defect. Patients with BAV frequently develop aortopathy, which depends on the dysfunction and morphotype of the BAV.

**Aim:**

The aim of our study was to compare the echocardiography and cardiac magnetic resonance (CMR) findings in BAV patients, and to define the risks of BAV dysfunction and aortopathy.

**Methods:**

The retrospective study included 50 patients (68% male) with BAV, with an average age of 13.6 ± 3.9 years, who underwent a transthoracic echocardiographic examination and CMR at our institute from 2012 to 2020.

**Results:**

The BAV types were evaluated significantly differently by echocardiography and CMR (*p* = 0.013). 54% of patients had BAV insufficiency on echo and 70% on echo CMR. It was more prevalent in males, older patients, and patients with a higher body surface area. By comparing the degree of insufficiency measured by echo (1+, IQR 0–1), and CMR (0, IQR 0–1), a significant difference was observed (*p* = 0.04), while a moderate positive correlation was proved (*rr* = 0.4; *p* = 0.004). Stenosis was registered in 44% of patients by echo, while 58% had stenosis on CMR. The peak pressure gradient measured by echo was significantly higher than the velocity on CMR (41, IQR 22.7–52.5 mmHg vs. 23, IQR 15.5–35.0 mmHg; *p* = 0.002). Aortopathy was registered in 76% of patients on echo and 78% on CMR; 38% of patients had severe aortic dilatation on echo and 54% on CMR (*p* = 0.003). Patients with BAV stenosis on echo had more frequent dilatation of the tubular ascending aorta (15/24 pts; *p* = 0.02). All patients with BAV insufficiency on CMR had aortopathy (*p* = 0.04) and had enlargement of the sinus of Valsalva and sinotubular junction. In patients with associated coarctation, the development of aortopathy occurred less frequently than those without coarctation (7/39 vs. 32/39; *p* = 0.003). The Bland-Altman method, a specific type of scatterplot that is used to visualize the results of comparing two measures, demonstrated the existence of agreement between the two methods, and a level of agreement between the methods of 95% was demonstrated.

**Conclusion:**

Our study indicated significant differences in the measured BAV morphotype and dysfunction when comparing the two diagnostic methods. On the other hand, moderate to strong correlations were found in the evaluated parameters, which indicates the importance of performing noninvasive diagnostic procedures in the follow-up of these patients.

## 1 Introduction

The bicuspid aortic valve (BAV) is the most common congenital heart defect (CHD) with male predominance (3:1). The prevalence of BAV ranges from 0.4% to 2.25%; about 0.5%–0.8% has been reported in healthy school children and young adults (1). Different authors described three or four different types of BAV, and they have been related to the progression of valve dysfunction or aortic dilation and the association with additional CHD (1–7).

The natural course of isolated BAV in both symptomatic and asymptomatic patients can be presented as aortopathy (about 50% of children), aortic stenosis (22%), aortic insufficiency (10%) and infective endocarditis (2%–3%). Children with isolated BAV and no or mild valve dysfunction at diagnosis usually have minimal disease progression before adulthood (1–3).

Bicuspid aortopathy is present in approximately 50% of patients with BAV (1, 2, 7). The development of aortic dilatation in patients with BAV is enigmatic. Two important theories have been previously described: (1) hemodynamic, and (2) genetic (8). The strongest predictors of progressive dilation of the proximal ascending aorta (AA) were severe aortic stenosis (AS) and moderate or severe aortic regurgitation (AR). In contrast, a prolonged aortic dilatation rate was reported in children with a normally functioning BAV (7, 9).

Echocardiography is most frequently used because it is widely available, affordable, and easy to use. In patients with BAV, morphotype is detected in 70%–90% with high sensitivity and specificity levels. However, image quality could be limited by poor acoustic windows, especially while imaging the aorta (10). Cardiovascular magnetic resonance (CMR) offers several benefits over ECHO, including more reliable and higher quality imaging and lack of dependence on patient proportions or acoustic windows (11). CMR is usually performed in older children and adolescents with dilation of the aortic root or ascending aorta. If the measurements are comparable with the ECHO values, then echocardiography can generally be used for follow-up. It is essential to point out that CMR underestimates the evaluation of the peak jet velocity due to its lower temporal resolution than echocardiography (12). On the other hand, CMR measurement of the largest aortic root dimension is more reliable than ECHO, especially when the root is asymmetric. Consequently, ECHO is the imaging technique of choice to diagnose BAV, valve morphotype, and valvular dysfunction in clinical practice. However, CMR is more reliable when assessing the aortic root and proximal AA (10, 11).

The primary aim of our study was to compare the echocardiography and CMR findings in patients with BAV. The secondary aim was to define our pediatric cohorts’ valve dysfunction and aortopathy risk factors.

## 2 Materials and methods

The retrospective cross-sectional study included 50 patients with BAV who underwent a transthoracic echocardiographic examination and CMR performed a maximum 3 months apart from 2012 to 2020 at the Mother and Child Health Institute of Serbia “Dr Vukan Čupić”. All echocardiographic and CMR parameters were re-measured for this study. The demographic parameters were analysed and compared with echocardiography and CMR findings.

### 2.1 Transthoracic echocardiography

Two pediatric cardiologists performed the echocardiography examinations on GE VIVID9 and PHILIPS EPIQ 7. The BAV type and function were evaluated. The diameters of all anatomical regions of the aorta (annulus, sinus of Valsalva (soV), sinotubular junction (STJ), and tubular AA) were measured by M-mode, 2-D echocardiography, and Detroit z-scores estimations.

Diagnosis is based on recognising a “fish-mouth” appearance of the aortic orifice in systole. The raphe represents the junction, the fusion of the cusps and often, on echocardiographic examination, can imitate the commissure so that the aortic valve looks like a tricuspid. According to Schafer et al., three anatomical morphotypes of BAV have been described. Type I with a fusion of the right and left coronary cusps (anterior-posterior); type II with the fusion of the right and non-coronary cusps (right-left); and type III with the fusion of the left and non-coronary cusps (13). We classified BAV without raphe in type 0.

Four different types were distinguished in the presence of flow disturbance registered on BAV: (1) functionally normal, (2) insufficient, (3) BAV stenosis, and (4) mixed dysfunction (insufficiency and stenosis).

Aortic insufficiency is divided into mild, moderate, and severe (Supplementary Table S1) (14).

Aortic stenosis is assessed in an apical long axis using continuous Doppler (CW), and the transvalvular pressure gradient is measured based on flow velocity at the level of the aortic orifice. According to the peak pressure gradient (PG), stenosis severity is divided into mild (PG < 30 mmHg), moderate (PG 31–60 mmHg), and severe (PG > 60 mmHg).

The dimensions of the aorta were estimated in the long parasternal axis using the inner edge technique (from one inner edge to the other inner edge of the aorta) at the height of the aortic annulus, soV, STJ and tubular AA. The absolute values of the dimensions of the heart cavities and the aorta are expressed by the score of standard deviations (Z score), which is evaluated relative to the child's body mass or body surface area (BSA). Values of Z score > 2 are qualified as dilation, and values of Z score > 4 are qualified as significant dilation.

### 2.2 Cardiac magnetic resonance

Cardiac MRI examinations were performed on a 1.5 T Siemens Avanto. A radiologist analysed the CMR findings. No sedative drugs were used for the CMR examination.

The aortic valve and aortic morphology were evaluated on cine steady-state free precession (SSFP). These sequences allow direct visualisation of the morphology of cusps and their number, and the direct planimetry of the valve area. It is useful for determining the type of bicuspid aortic valve.

Aortic measurements were made in a cross-sectional plane, inner edge to inner edge, using double oblique 2D cine SSFP (three chambers and left ventricular outflow tract views) in systole. Evaluation of the aorta included the aortic valve annulus, soV (aortic root), STJ, tubular AA (at the level of pulmonary bifurcation), distal transverse aortic arch, aortic isthmus, and descending aorta. The geometry of the aortic root can be asymmetric, especially in patients with dilatation and abnormal aortic valve morphology. Measuring the largest dimensions within the aortic root is recommended: the largest sinus-to-sinus diameter and the largest commissure-to-sinus diameter. Z scores measurements were based on MR angiography-derived normative values provided by Kaiser et al. (15).

Phase contrast pulse sequences with through-plane velocity encoding were performed for functional evaluation of the aortic valve. In this sequence, peak flow velocity, peak and medium pressure gradient, forward and regurgitant flow, and regurgitant fraction were assessed. CMR-derived regurgitant fraction cut-offs are mild-AR (<20%), moderate AR (20%–40%), and severe AR (≥40%) (15). The peak jet velocity of more than 3 m/s was considered moderate AS, while more than 4.0 m/s was classified as severe AS (12, 16, 17).

Cardiac MRI also provides morphologic and functional data of the whole heart, and evaluation of abnormalities usually associated with a bicuspid aortic valve. It is instrumental to obtain repeated measurements during the patient's follow-up.

### 2.3 Statistical analysis

Data was processed using statistical software SPSS 25.0 for Windows 10. Among the descriptive statistical methods, measures of central tendency (arithmetic mean, median, mode), measures of variability (standard deviation) and relative numbers (structure indicators) were used. The difference in the distribution of specific parameters among the studied groups was determined using *χ*^2^ or Fisher's test. Shapiro–Wilk and Kolmogorov–Smirnov tests were used to assess the normality of the distribution of numerical variables. Groups were compared using the Student's *t*-test and ANOVA test for independent data for numerical variables with a normal distribution. In contrast, the Mann–Whitney test and Kruskal Wallis were used for numerical variables with a non-normal distribution. The Paired *t*-test and Wilcoxon test were used to compare two dependent samples. Pearson or Spearman tests were used to determine correlations between the parameters in different groups.

The Bland-Altman method was used to examine the agreement between the two methods to define the concordance interval. The Bland-Altman (mean-difference or limits of agreement) plot and analysis are used to compare two measurements of the same variable. So, it is a method comparison technique.

All the statistical methods were considered statistically significant, *p* ≤ 0.05.

## 3 Results

The study included 50 patients (68% male) with an average age of 13.6 ± 3.9 years. Isolated BAV was registered in 28 patients. The combination of BAV and coarctation of the aorta (CoA) was registered in 28% of patients, and BAV and dysplasia of the aortic arch in 4%. Patients with CoA had type 1 BAV more frequently (12/14; *p* = 0.04) than patients without CoA.

### 3.1 Bicuspid valve morphotype and dysfunction

The BAV morphotypes were evaluated significantly differently by echocardiography and CMR (*p* = 0.013). The echocardiographic and CMR findings indicated that 78% of patients had type I BAV, while type II BAV was registered in 16% by echo and 22% by CMR. By comparing the aortopathy frequency rate assessed by echocardiography (76%) and CMR (78%), there is no difference between the use of these two diagnostic methods (Table 1).

**Table 1:** The frequency of BAV types, function and aortopathy considering two different methods.

parameter	ECHO	CMR	*p*-value
	Frequency (%)	Frequency (%)	
BAV	48 (96)	50 (100)	1.0
Type BAV			
0	2 (4)	39 (78)	**0** **.** **013**
1	39 (78)	11 (22)	
2	8 (16)		
3	1 (2)		
BAV insufficiency	27 (54)	15 (30)	**<0** **.** **001**
Insufficiency severity			
0	23 (46)	35 (70)	**0** **.** **03**
1	24 (48)	10 (20)	
2	2 (4)	4 (8)	
3	1 (2)	1 (2)	
BAV stenosis	22 (44)	29 (58)	**0** **.** **01**
Stenosis degree			
0	28 (56)	21 (42)	**0** **.** **005**
1	9 (18)	19 (38)	
2	8 (16)	9 (18)	
3	5 (10)	1 (2)	
Aortopathy	38 (76)	39 (78)	1.0
Dilatation			
soV	28 (56)	29 (58)	0.7
STJ	22 (44)	23 (46)	
AoAsc	24 (48)	28 (56)	

BAV, bicuspid aortic valve; soV, sinus of Valsalve; STJ, sinotubular junction; AoAsc, ascending aorta.

Bold values are statistically significant.

BAV insufficiency was more frequently observed by echo than CMR (*p* < 0.001; Table 1). 54% of patients had BAV insufficiency on echo, 24 had mild, 2 patients had moderate, and 1 patient had severe. Insufficiency was more common in males than females (22/27 males vs. 5/27 females with insufficiency; *p* = 0.027).

Patients with BAV insufficiency had higher BSA (1.7 ± 0.3 m^2^) compared to patients without insufficiency (1.4 ± 0.4 m^2^; *p* = 0.004). Those patients (with insufficiency) were older than the others (16 years, IQR 12–18, vs. 13, IQR 10–16; *p* = 0.02). Additionally, a moderate positive correlation was proved between the degree of insufficiency (ECHO and CMR) and patient age (*rr* = 0.32; *p* = 0.02), and the degree of insufficiency and BSA (*rr* = 0.4; *p* = 0.04). On the other hand, CMR findings showed that 70% of patients did not have BAV insufficiency, while mild insufficiency was in 20%. The degree of insufficiency significantly differed depending on the diagnostic methods used (*p* = 0.03) (Table 1). By comparing the degree of insufficiency measured by echo (1+, IQR 0–1), and CMR (0, IQR 0–1), a significant difference was observed (*p* = 0.04), while a moderate positive correlation was proved (*rr* = 0.4; *p* = 0.004).

Patients with type I BAV on CMR had mild insufficiency more frequently than type 2, while patients with type 2 BAV who developed aortic insufficiency had moderate-severe regurgitation (*p* = 0.03) (Table 2). BAV type did not influence BAV function (*p* = 0.4).

**Table 2:** The difference in the degree of BAV insufficiency regarding the BAV types (absolute value).

	Without	Mild	Moderate- severe	*p*-value
Type I	27	10	2	**0** **.** **03**
Type II	8	0	3	
All	35	10	5	

Bold values are statistically significant.

44% of patients had BAV stenosis registered by echo, while 58% had stenosis on CMR (*p* = 0.01). Mild stenosis was markedly more frequent on CMR (*p* = 0.005)—in 9 patients on echo and 19 patients on CMR, while five patients had severe stenosis on echo and one on CMR (*p* = 0.005) (Table 1). The peak PG measured by echo was significantly higher than by CMR (41 mmHg, IQR 22.7–52.5 mmHg vs. 23 mmHg, IQR 15.5–35.0 mmHg; *p* = 0.002).

### 3.2 Bicuspid valve aortopathy

By comparing the aortopathy frequency rate assessed by echo (76%) and CMR (78%), there is no difference between the use of these two diagnostic methods (Table 1). Gender and age did not influence the type of aortopathy. Severe aortic dilatation was observed in 38% and 54% of patients by echo and CMR, respectively (*p* = 0.003).

The CMR findings indicated that all patients with type 2 BAV had aortopathy (11/11; *p* = 0.04). Severe aortic dilatation was found in 6/11 patients with type 2 and 21/39 patients with type 1. The patients with type 2 BAV had a dilated STJ more often (8/11 patients; *p* = 0.04), while patients with type 1 had more frequent dilatation of the tubular AA on CMR (*p* = 0.03).

All patients with BAV insufficiency had aortopathy (*p* = 0.04) and more frequent STJ dilatation (15/22; *p* = 0.07). On the other hand, patients with BAV stenosis on echo had more frequent dilatation of the tubular AA (15/24 pts; *p* = 0.02). The degree of insufficiency and stenosis did not influence the thoracic aorta diameter Z score. It was also shown that patients with functionally normal BAV also have aortopathy (*p* = 0.01) (Table 3). Those patients had dilatation of the soV and STJ more often on CMR (Table 4) with a statistically significantly higher Z score of soV (*p* = 0.005) and STJ (*p* = 0.05) (Supplementary Figure S1).

**Table 3:** The frequency of aortopathy depends on the type of BAV defect.

Type (ECHO)	Aortopathy		*p* value
	No	Yes	
Normal	2	17	**0** **.** **01**
Insufficiency	8	6	
Stenosis	0	8	
Insuf + sten	11	8	

Insuf, insufficiency; sten, stenosis.

Bold values are statistically significant.

**Table 4:** Difference in Z scores of aortic diameters depending on the type of BAV defect.

	Z score annulus	Z score bulbus	Z score STJ	Z score AoAsc
Normal	2.4 (IQR 1.5–3)	4.3 (IQR 2.2–5.1)	2.8 (IQR 1.1–6)	2.5 (IQR 1.3–5.4)
Insufficiency	1.8 (IQR 0.7–2.8)	4.3 (IQR 1–6)	1.9 (IQR 1.6–5.9)	1.3 (IQR 0.5–5.3)
Stenosis	1 (IQR 0.1–2.2)	0.8 (IQR 0.2–2.4)	0.6 (IQR 0–2)	1.9 (IQR −0.05–3.6)
Insuf + sten	2.4 (IQR 0.5–4.5)	2.6 (IQR −0.2–4.9)	1.9 (IQR −0.5–3.5)	4.2 (IQR 3.6–5.4)
*p*-value	0.3	**0** **.** **02**	**0** **.** **02**	0.4

Insuf, insufficiency; sten, stenosis; STJ, sinotubular junctions; AoAsc, ascending aorta.

Bold values are statistically significant.

In patients with associated CoA, aortopathy developed less frequently than in those without CoA (7/39 vs. 32/39; *p* = 0.003). In this cohort of patients, the Z score of all anatomical parts of the AA was lower (Table 5).

**Table 5:** Echocardiography and CMR evaluate differences in Z scores of the aorta's anatomical domains depending on its coarctation.

	ECHO		*p* value	CMR		*p* value
	CoA			CoA		
	No	Yes		No	Yes	
Z score annulus	2.6 1.2–3.4	2.1 0.7–2.7	0.4	1.9 0.8–2.8	2.1 1–3.3	0.5
Z score bulbus	2.8 1.5–3.7	1.9 0.5–2.3	**0** **.** **05**	4 1.1–5.3	1.7 0.3–2.3	**0**.**01**
Z score STJ	2 1.3–3.6	1.7 0.7–2.4	0.2	2,3 0.6–4.8	1.7 0–2	**0**.**05**
Z score AoAsc	2.2 1.2–4.4	1 0.3–2.5	**0**.**05**	2.8 1.7–6.4	1.7 0.5–3.1	**0**.**05**

STJ, sinotubular junction; AoAsc, ascending aorta; CoA, aortic coarctation.

Bold values are statistically significant.

**Figure 1:**
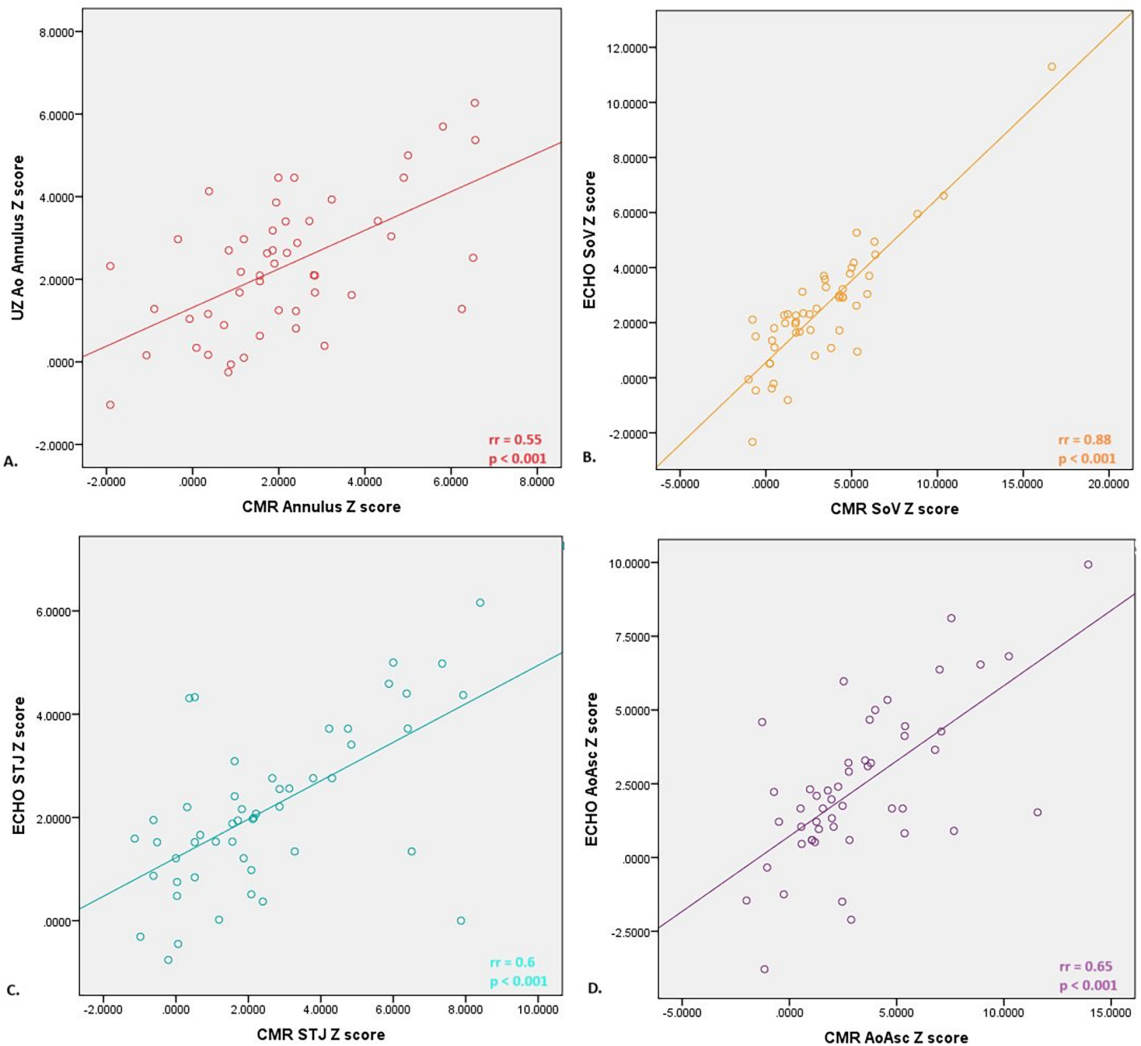
Correlation between Z scores of diameters of different anatomical domains of the aorta measured by echocardiography and nuclear magnetic resonance of the heart; **(A)** moderate positive correlation was proved between aortic annulus diameter evaluated by echo and CMR< **(B)** strong positive correlation was proved between soV (bulbus) diameter evaluated by echo and CMR; **(C)** moderate positive correlation was proved between STJ diameter evaluated by echo and CMR; **(D)** moderate positive correlation was proved between aoAsc diameter evaluated by echo and CMR. STJ, sinotubular junction; AoAsc, ascending aorta.

**Figure 2:**
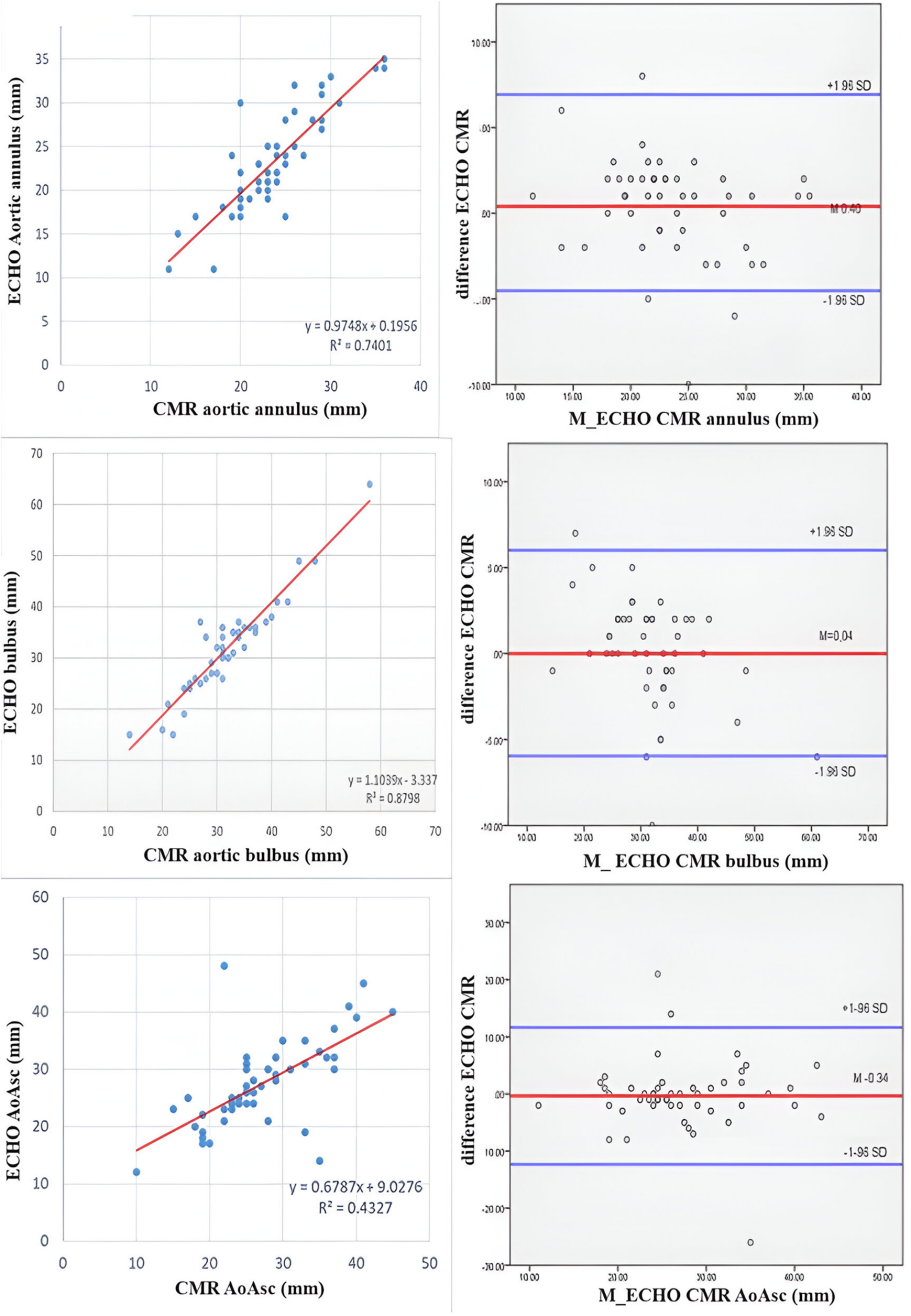
Bland-Altman plot showing concordance between aortic diameters measured by echocardiography and CMR. The *X*-axis represents the mean of the 2 measures taken for each patient, whereas the *Y*-axis represents the arithmetical difference between the 2 measures. Each measurement is plotted on the graph, yielding visualization of how similar the 2 measurement techniques are (*Y*-axis) across the range of measurement values (*X*-axis). In addition to the *X* axis, 3 horizontal lines are plotted: the middle line represents the mean difference between the 2 measurements (ideal value is 0), whereas the upper and lower lines represent the 95% limits of agreement between the 2 measurement techniques.

A comparison of the Z scores obtained by echocardiography and CMR did not show the existence of a statistically significant difference, except in the Z score of the diameter of the aortic arch (ECHO 1.3; IQR 0.5–1.8 vs. CMR −0.5; IQR −2.1–2.55, *p* = 0.03). A strong positive correlation was shown between the echocardiographic and CMR parameters (Figure 1). The Bland-Altman method demonstrated the existence of agreement between the two methods, and a level of agreement between the methods of 95% was demonstrated (Figure 2).

## 4 Discussion

While echocardiography has been the traditional modality for imaging patients with BAV, CMR has emerged in the last decade as a powerful modality. By literature review, we did not find comparative studies between CMR and ECHO in evaluating pediatric patients with BAV. Echocardiography, as a more accessible method, represents an accurate and reliable method for evaluating the function and morphology of BAV and BAV aortopathy, which allows the need for CMR to be optimised in time.

We found that the BAV morphotypes were evaluated significantly differently when comparing echocardiography and CMR findings, similar to another study (18). Namely, the identification of a raphe could be complicated by echocardiography (6). The frequency of BAV insufficiency evaluated by echocardiography was higher, while BAV stenosis was more frequent on CMR. Although a positive correlation was proved, we found that the echocardiography degree of insufficiency was higher than the degree of insufficiency on the CMR finding. Our study showed that CMR underestimates the degree of BAV stenosis compared to echocardiography. It could be explained secondary to intravoxel dephasing errors in the presence of high-velocity flows (16, 17).

We found a similar frequency of BAV aortopathy estimated by echocardiography and CMR, but a difference was found in the severity of dilatation of the thoracic aorta. Consequently, CMR provides more precise information than echocardiographic examination. We found a strong positive correlation between the annulus, soV and STJ Z scores estimated by echocardiography and CMR. Wenzel et al. found that CMR measurements of the aortic root were comparable to echocardiographic measurements with excellent reproducibility (19). Still, the main difference was found in the tubular AA and aortic arch. The Bland-Altman method, a specific type of scatterplot that is used to visualize the results of studies comparing 2 measures, demonstrated the existence of an agreement between the two methods, and a level of agreement between the methods of 95% was demonstrated. The study conducted by Wenzel showed similar results to ours (19).

### 4.1 Bicuspid valve morphotype

In a MIBAVA (Mechanistic Interrogation of BAV-associated Aortopathy) Consortium large multicentre retrospective study, which involved 2,122 children with BAV (average age 10.2 years), the most common morphotype was type 1 (65.7%), followed by type 2 (32.9%) (7). In our study, 78% had type 1 BAV, and this morphotype was more frequent in patients with CoAo. Grattan et al. found that R-L type was more frequent in patients with left heart obstructive lesions and CoAo. At the same time, R-N fusion was associated with valve dysfunction and required more interventions (7). Our patients with R-L fusion had more common mild BAV insufficiency, while patients with type 2 BAV had no valve dysfunction. Still, if they developed aortic insufficiency, it was moderate-severe regurge.

### 4.2 Bicuspid valve dysfunction

Aortic regurgitation is typically secondary to thickening and progressive immobility of AV leaflets (2). 54% of patients had BAV insufficiency on echo and 70% on echo CMR, and most of the patients had mild insufficiency. Fernandes et al. described mild AR in 33% of patients in the study, while moderate or more AR was observed in only 4.5% of patients, and these children were older than the remaining patients (5). These statements were similar to our study. Namely, the degree of insufficiency correlated positively with patient age and BSA. Additionally, we found that males had insufficiency more frequently than females.

AS develops and progresses slowly due to a disproportion in the increase in cardiac output and the absence of growth in the effective opening of BAV (2). On echocardiography examination, 44% of patients had BAV stenosis, while 10% had severe stenosis. On the other hand, CMR indicated that 58% of patients had stenosis, and only one had severe stenosis. In a MIBAVA Consortium study, any aortic stenosis was observed in 35.2% of patients, and severe stenosis was observed in 10.5% (7). Fernandes et al., in a retrospective review of 1,135 patients with BAV, identified moderate or greater AS on the initial echocardiograms in 9.7% of patients with RL cusp fusion, and 25.9% with RN cusp fusion (3). BAV morphotype did not influence the BAV stenosis evaluated by echo and CMR in our study.

### 4.3 Bicuspid valve aortopathy

Patients with BAV frequently demonstrate dilation of the aorta, even as children and adolescents, with or without BAV dysfunction (1–8). Aortopathy was observed in 76% and 78% of patients on echo and CMR, respectively, which was greater compared to the other study. The severity of aortic dilatation differed significantly between the two diagnostic methods. On echocardiography, severe aortic dilatation had 38% of our patients, while on CMR 54%. In a MIBAVA study, almost 50% had aortic dilatation, while 9.1% had severe dilatation (7). The difference in the aortopathy frequency could be explained by the fact that CMR is usually performed in patients with suspected BAV aortopathy and severe valvular dysfunction, which was the inclusion criteria for our study.

Valve dysfunction and BAV morphotype influenced the aortopathy phenotype. Namely, we found that all patients with type 2 BAV had aortopathy, and most of them had severe aortic dilatations. Additionally, patients with type 2 BAV had more frequent dilatation of STJ. On the other hand, patients with type 1 BAV had more frequent dilatation of tubular AA. This finding was the opposite of the results in the MIBAVA Consortium study. This study showed that patients with R-L fusion were associated with a larger soV, while R-N fusion was associated with a larger tubular AA (7). The data from the study included 4-dimensional MRI flow, suggesting that even without AS, BAV type 2 results in significant flow disturbance in the AA. Consequently, the independent association between type 2 BAV and tubular AA dilatation may be related to low-velocity flow disturbances (2).

Valve dysfunction had a significant influence on aortic dilatation. Studies in children and adolescents indicated AR was associated with a larger soV and tubular AA (1, 3, 7). We found that patients with BAV insufficiency had more frequent STJ dilatation on echocardiography, but the degree of insufficiency did not influence dilatation severity. Additionally, we found that those patients and patients with normally functioning BAVs had more frequent dilatation of the soV, which was similar to other studies. Those findings suggested a genetic predisposition to developing this type of root aortopathy (7, 9). In patients with isolated BAV stenosis, dilatation of tubular AA was more common. This statement agreed with findings from other studies and supported the hemodynamic hypothesis in developing this ascending phenotype of BAV aortopathy (7). The degree of stenosis did not influence the severity of aortopathy. In adolescents with combined valve dysfunction, dilatation of the aortic annulus, soV and ascending aorta was observed. Such a finding in our patients supports the claim that genetic predisposition and hemodynamic influence play a role in developing this type of combined aortopathy (4, 6, 7).

Spaziani et al. found that in patients with aortic coarctation, aortic diameters were smaller than the BAV group (4). At the same time, Gratten et al. showed that the absence of CoA was positively associated with dilatation of the aortic sinuses and ascending aorta (7). We proved that adolescents with CoA, except for mildly dilated aortic annulus, had normal aorta diameters that were significantly smaller than those of patients without coarctation. The association between CoA and aortic dilatation in patients with BAV has not been well defined (4, 5, 7).

The major limitations were the retrospective design and the number of patients for the final conclusion and recommendations. Consequently, further multicenter studies with the most prominent number of pediatric patients should be conducted.

## 5 Conclusion

Our study indicated that although differences between ECHO and CMR exist, these two diagnostic methods are comparable and complementary. Namely, a difference in interpretation was found between BAV morphotype and function but not between the rate and type of aortopathy. Type 2 BAV was more commonly registered on CMR. The BAV stenosis was more commonly found on CMR than ECHO, but the CMR underestimated the degree of aortic stenosis.

The difference between the aortopathy revealed by CMR and ECHO was not found, but the severe aortopathy was commonly found on CMR. Although a strong positive correlation exists, CMR rated the severity of aortic dilatation more precisely. All patients with type 2 BAV and insufficiency had aortopathy. Patients with type 1 had predominantly tubular AA dilatation, while patients with type 2 had STJ dilatation. On the other hand, BAV insufficiency produced dilatation of STJ, but stenosis is related to tubular AA dilatation. Patients with CoA had more frequent type 1 BAV and did not have aortopathy.

Our results suggest that in patients with BAV, careful follow-up by echocardiography is necessary to assess valve function and the existence of aortopathy, and CMR should be performed in adolescents to obtain the most accurate information about the BAV morphotype and the severity of aortopathy.

## Data Availability

The original contributions presented in the study are included in the article/Supplementary Material, further inquiries can be directed to the corresponding author.

## Supplementary material

Supplementary Figure S1 Graphic display of Z scores of the soV and STJ assessed by CMR depending on the degree of BAV stenosis. CMR, cardiac magnetic resonance; soV, sinus of Valsalve; STJ, sinotubular junction; BAV, bicuspid aortic valve.
