# Supplementary material for: Differences between echocardiography and cardiac nuclear magnetic resonance parameters in children with bicuspid aortic valve-related aortopathy
Source: Front Cardiovasc Med. 2024 Nov 26;11:1384707. doi: 10.3389/fcvm.2024.1384707 (PMC11629474; doi:10.3389/fcvm.2024.1384707)
Supplement: Supplementary Figure S1 — Graphic display of Z scores of the soV and STJ assessed by CMR depending on the degree of BAV stenosis. CMR, cardiac magnetic resonance; soV, sinus of Valsalve; STJ, sinotubular junction; BAV, bicuspid aortic valve. [file Table1.docx]

Supp. Table 1. Grading of aortic regurgitation by echocardiography

| AR severity | mild | moderate | severe |
| --- | --- | --- | --- |
| Colour flow AR jet width | The end of the proximal part of the AMC | The end of the distal part of the AMC | To the middle or end of LV |
| PHT | >500 ms | 500-200 ms | <200 ms |
| Vena contracta | < 3 mm | 3-6 mm | >6 mm |
| Relation between AR jet width and diameter LVOT | <25% | 25-65% | >65% |
| Left ventricle dilatation | No | mild | moderate - severe |

Abbreviations: PHT – pressure half time, AR – aortic regurgitation; LV – left ventricle; AMC – anterior mitral cusp
