# Supplementary figures and images for: Differences between echocardiography and cardiac nuclear magnetic resonance parameters in children with bicuspid aortic valve-related aortopathy
Source: Front Cardiovasc Med. 2024 Nov 26;11:1384707. doi: 10.3389/fcvm.2024.1384707 (PMC11629474; doi:10.3389/fcvm.2024.1384707)

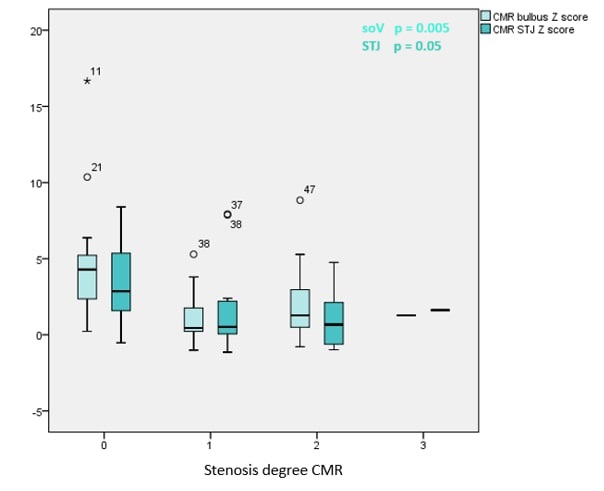

Supplement: Supplementary file 2 [file Image1.jpeg]
